# Brain Activity after Intermittent Hypoxic Brain Condition in Rats

**DOI:** 10.3390/brainsci12010052

**Published:** 2021-12-30

**Authors:** Bora Mun, Yun-Chol Jang, Eun-Jong Kim, Ja-Hae Kim, Min-Keun Song

**Affiliations:** 1 Department of Physical & Rehabilitation Medicine, Chonnam National University Medical School & Hospital, Gwangju 61469, Korea; bora1604@daum.net (B.M.); with-you85@hanmail.net (Y.-C.J.); trueisone@daum.net (E.-J.K.); 2 Department of Nuclear Medicine, Chonnam National University Medical School & Hospital, Gwangju 61469, Korea; jhbt0607@hanmail.net

**Keywords:** intermittent hypoxia, hypoxic-ischemic brain injury, brain activity, angiogenesis

## Abstract

Hypoxic brain injury is accompanied by a decrease in various functions. It is also known that obstructive sleep apnea (OSA) can cause hypoxic brain injury. This study aimed to produce a model of an intermittent hypoxic brain condition in rats and determine the activity of the brain according to the duration of hypoxic exposure. Forty male Sprague–Dawley rats were divided into four groups: the control group (*n* = 10), the 2 h per day hypoxia exposure group (*n* = 10), the 4 h per day hypoxia exposure group (*n* = 10), and the 8 h per day hypoxia exposure group (*n* = 10). All rats were exposed to a hypoxic chamber containing 10% oxygen for five days. Positron emission tomography–computed tomography (PET-CT) brain images were acquired using a preclinical PET-CT scanner to evaluate the activity of brain metabolism. All the rats were subjected to normal conditions. After five days, PET-CT was performed to evaluate the recovery of brain metabolism. Western blot analysis and immunohistochemistry were performed with vascular endothelial growth factor (VEGF) and brain-derived neurotrophic factor (BDNF). The mean SUV was elevated in the 2 h per day and 4 h per day groups, and all brain regions showed increased metabolism except the amygdala on the left side, the auditory cortex on the right side, the frontal association cortex on the right side, the parietal association cortex on the right side, and the somatosensory cortex on the right side immediately after hypoxic exposure. However, there was no difference between 5 days rest after hypoxic exposure and control group. Western blot analysis revealed the most significant immunoreactivity for VEGF in the 2, 4, and 8 h per day groups compared with the control group and quantification of VEGF immunohistochemistry showed more expression in 2 and 4 h per day groups compared with the control group. However, there was no significant difference in immunoreactivity for BDNF among the groups. The duration of exposure to hypoxia may affect the activity of the brain due to angiogenesis after intermittent hypoxic brain conditions in rats.

## 1. Introduction

Hypoxic brain injury in 30–50% of children is known to be accompanied by a developmental delay with neurologic symptoms [1], and patients with severe bleeding during surgery of, for example, the heart or aortae also often show hypoxic-ischemic brain damage and a decline in neurological function even after recovery [2].

Intermittent hypoxic exposure from obstructive sleep apnea syndrome may induce cognitive decline because of neuronal cell apoptosis in the cortex and hippocampus [3,4,5]. Cognitive decline, especially spatial memory, after intermittent hypoxic exposure was reported in several studies [6,7,8,9,10,11]. Functional deficits were also reported and the mechanism triggering these impairments was suggested to be related to neuronal apoptosis and oxygen-free radicals, which may activate a harmful process under a chronic intermittent hypoxic condition [12,13]. However, some studies reported that hypoxic conditioning may protect subjects from neurodegenerative disease and have a treatment effect for psychiatric disease [14,15,16,17]. In an epilepsy rat model, both hypoxic preconditioning and postconditioning circumstances may reduce the apoptotic neuronal cells in the hippocampus and help to improve spatial memory [18]. Moreover, a hypoxic condition may promote a neuroprotective effect in several cerebral diseases such as ischemic stroke, Alzheimer’s disease, psychiatric diseases, or seizures. However, the duration of intermittent hypoxic exposure that affects neurologic function is not known. The spontaneous recovery threshold according to the exposure duration has also not yet been reported. 

This study aimed to produce a model of an intermittent hypoxic brain condition in rats and to determine the activity and mechanism of brain metabolism after exposure to various durations of intermittent hypoxia in rats. 

## 2. Materials and Methods

### 2.1. Experimental Subjects

The experimental subjects were eight-week-old male Sprague–Dawley rats (Samtako Co., Osan, Korea) weighing 300 ± 50 g. All subjects were housed in regular circumstances at the University Animal Care Laboratory. The study protocol was approved by the Institutional Animal Ethics of the University Animal Care and Committee (CNUH IACUC-18018) and all experimental procedures followed the guidelines of the IACUC.

A total of 40 rats were randomly separated into four groups: control group (*n* = 10), 2 h per day hypoxia exposure group (*n* = 10), 4 h per day hypoxia exposure group (*n* = 10), and 8 h per day hypoxia exposure group (*n* = 10).

### 2.2. Methods

#### 2.2.1. Hypoxic Brain Injury Rat Model

**Figure 1:**
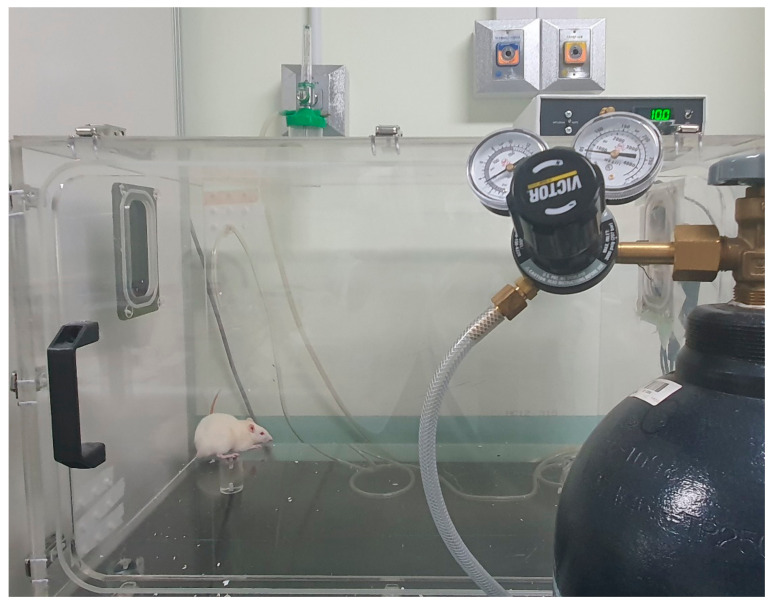
Hypoxic chamber with 10% oxygen. Animals sojourned for 2 (*n* = 10), 4 (*n* = 10), and 8 h per day (*n* = 10) in one identical commercially designed chamber (30 × 320 × 320 inches) for five days. Deviations from the desired concentration were corrected by the addition of N_2_ through solenoid valves.

The hypoxic injury rat model was induced with a hypoxic chamber (Figure 1). The animals stayed for 2 h per day (*n* = 10), 4 h per day (*n* = 10), and 8 h per day (*n* = 10) in one identical commercially designed chamber (30 × 320 × 320 inches) over five days under a 10% oxygen concentration, while the control group was exposed to a normal oxygen concentration. Deviations from the desired concentration were corrected by the addition of N_2_ through solenoid valves. The humidity was measured and maintained at 40–50% by circulating the gas through a freezer and silica gel. The ambient temperature was kept at 22–24 °C. After each group was exposed to its condition for five days, all rats were then kept under normal circumstances in the cage with a normal oxygen concentration for five days to assess their spontaneous recovery pattern.

#### 2.2.2. Small-Animal Positron Emission Tomography–Computed Tomography Imaging Protocol

The positron emission tomography–computed tomography (PET-CT) brain images were acquired using a preclinical PET-CT scanner (Sedecal, Madrid, Spain). The animals were fasted for 6 h before the PET scans. The static PET data were acquired at 30 min after tail vein injection of [18F] FDG (fluorodeoxyglucose) with about 18.5 MBq (range: 12.25~25.38 MBq, mean: 19.21 MBq, SD: 2.86 MBq). After the 20 min PET scan, a CT study was acquired for attenuation correction. PET image reconstruction was carried out using the 3D ordered-subset expectation maximization (3D-OSEM) algorithm. 

**Figure 2:**
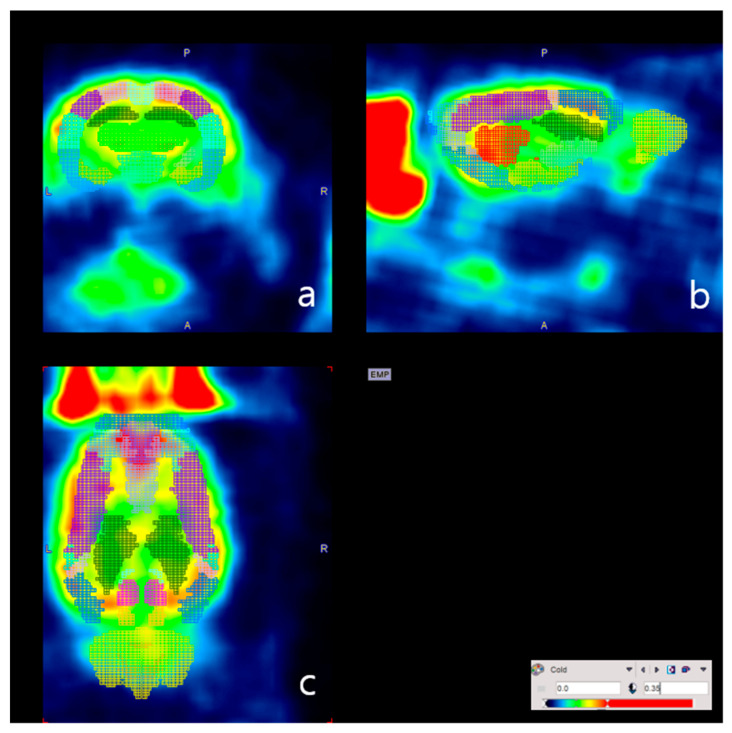
Quantification of FDG brain uptake of each cerebral region. Representative images-co-registered PET atlas images for one subject-are shown in the (**a**) coronal, (**b**) sagittal, and (**c**) axial orientations.

The PET-CT scan was performed twice. The first scan was done one day after exposure to 10% O_2_ concentration for five days. The second scan was carried out one day after normal circumstances for five days. The spherical volumes of interest (VOI) with a 6 mm diameter were drawn in both the cerebral hemispheres, covering both the cortex and subcortex of the rat brain, on VivoQuant^TM^ software. The mean standardized uptake values (SUVs) were measured and averaged, both of the left and right cerebral hemispheres. To quantify the FDG brain uptake (%ID/g) of each cerebral region (shown in Table 1), each PET image was transformed into the space of the PMOD (v4.1, PMOD Technologies, Switzerland) FDG rat brain template (W. Schiffer) (Figure 2).

#### 2.2.3. Western Blot Analysis

Western blot analysis was performed according to our laboratory protocol [19]. After the second PET-CT scan, all rats were sacrificed. The hippocampus was extracted from each rat brain. Hippocampus tissues (20 μg of each sample) were selected for Western blot analysis for the vascular endothelial growth factor (VEGF) and brain-derived neurotrophic factor (BDNF) proteins. The membranes were reacted with the primary antibody against rabbit VEGF (1:500, Novus Biologicals, Littleton, CO, USA) or rabbit BDNF (rabbit polyclonal antibody, 1:1000 dilution, Santa Cruz Biotechnology, Santa Cruz, CA, USA). The membranes were incubated in a secondary horseradish peroxidase-conjugated goat anti-rabbit IgG (1:1000 dilution, Upstate Biotechnology Inc., Lake Placid, NY, USA). Immunoreactive bands were displayed by enhanced chemiluminescence using the Immobilon Western Chemiluminescent substrate (Millipore, Billerica, MA, USA). Then, the protein bands were photographed in grayscale at 600 dpi and quantified using the Image J program (NIH, Bethesda, MD, USA) [20,21].

#### 2.2.4. Immunohistochemistry

Immunohistochemistry was performed according to our laboratory protocol [19]. After the second PET-CT scan, all rats were sacrificed. We focused on the hippocampal area which is well known for the most sensitive to hypoxic damage [22]. Hippocampus tissues were used to assess the reactivity of VEGF and BDNF. Briefly, they were incubated with the primary antibody, anti-VEGF (1:300 dilution; Novus Biologicals, Littleton, CO, USA) or anti-BDNF (1:200 dilution; Santa Cruz Biotechnology, Santa Cruz, CA, USA). The slides were reacted with the secondary rabbit anti-mouse IgG antibody (1:500, Chemicon, Billerica, MA, USA). The slides were counterstained with Mayer’s hematoxylin, scanned using an Aperio CS2 digital pathology slide scanner (Leica Biosystems, Wetzlar, Germany) and analyzed using Aperio Image Scope software (Leica Biosystems, Wetzlar, Germany). We also performed quantification of immunohistochemistry. Immunochemically stained tissues were quantified using the IHC image analysis toolbox developed based on Image J. The color and density of the tissue positive DAB were learned from the detected color through the program, and the background pixel was removed. After that, positive color pixels were selected and quantified based on the histogram [23,24].

#### 2.2.5. Imaging of Brain Activity

The micro PET-CT imaging at 5 days rest after hypoxia were displayed as color overlays on each immunohistochemistry scan at the level of hippocampus with the Adobe photoshop (Adobe photoshop 2020, Adobe Inc., San Jose, CA, USA) program 

#### 2.2.6. Statistical Analyses

Statistical analyses were conducted with SPSS for Windows (version 25.0, Chicago, IL, USA). Data were shown as the mean ± standard deviation (SD). One-way ANOVA was used to analyze the SUV mean along with the exposure duration. The Mann–Whitney U test was used to assess the association of brain region SUVs with hypoxic exposure times and a paired t-test was used for subgroup analysis. One-way ANOVA with LSD post hoc test was used to analyze the quantification of immunohistochemistry. The differences were regarded as statistically significant when the *p*-value was <0.05.

## 3. Results

### 3.1. Brain Metabolism Using Small-Animal PET/CT Imaging

**Figure 3:**
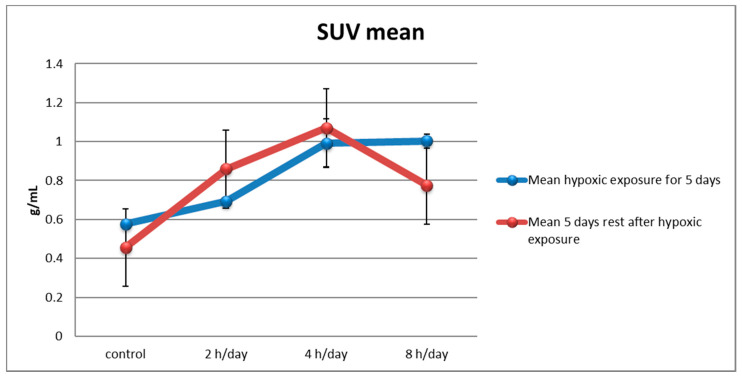
Brain metabolism using small-animal PET/CT imaging. After exposure to 10% O_2_ for one week, the mean SUV was elevated in the 4 and 8 h per day groups compared with the control group (*p* = 0.000). Next, all rats were subjected to normal conditions for one week; the mean SUV was elevated in the 2 4 h per day groups compared with the control group (*p* = 0.047). The mean SUV was decreased in the group with an exposure time of 8 h per day.

**Figure 4:**
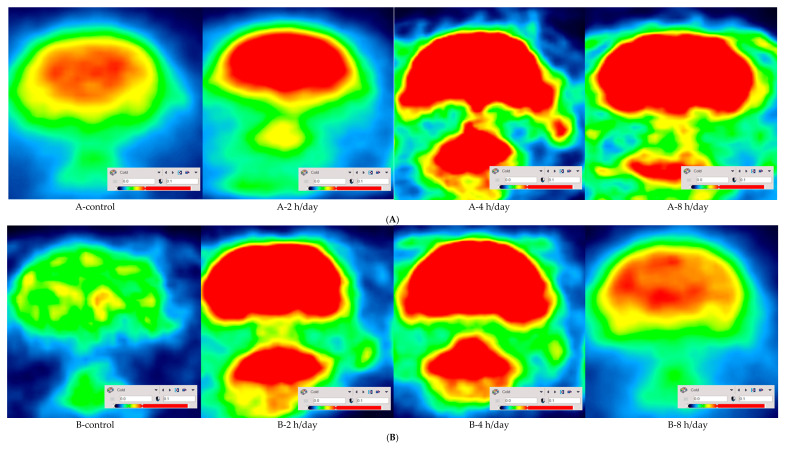
Micro PET-CT imaging after five days hypoxic exposure and at 5 days rest after hypoxia at the level of hippocampus. Activity of brain metabolism increased in 2, 4 and 8 h per day group compared with the control group after five days hypoxic exposure (**A**), however, Activity of brain metabolism increased in 2 and 4 h per day group compared with the control group at 5 days rest after hypoxia (**B**).

After exposure to 10% O2 concentration for five days, the mean SUV, which is averaged mean SUV of each hemispheres, was 0.576 ± 0.010 g/mL in the control group, 0.692 ± 0.020 g/mL in the 2 h per day group, 0.992 ± 0.124 g/mL in the 4 h per day group, and 1.002 ± 0.035 g/mL in the 8 h per day group. With one way ANOVA, the mean SUV was elevated in the 4 and 8 h per day groups compared with the control group (*p* = 0.000) after hypoxic exposure. The next day, after the first PET-CT scan, all rats were returned to normal circumstances for five days. The mean SUV was 0.456 ± 0.013 g/mL in the control group, 0.859 ± 0.353 g/mL in the 2 h per day group, 1.070 ± 0.097 g/mL in the 4 h per day group, and 0.776 ± 0.265 g/mL in the 8 h per day group. With one way ANOVA, the mean SUV was elevated in the 2 and 4 h per day groups compared with the control group (*p* = 0.047) after one week’s rest (Figure 3). These results may be represented by the micro PET-CT scan image (Figure 4).

The metabolism in each brain region was also analyzed. When we compared the hypoxic exposure groups with the control group, all brain regions showed increased metabolism except the amygdala on the left side, the auditory cortex on the right side, the frontal association cortex on the right side, the parietal association cortex on the right side, and the somatosensory cortex on the right side immediately after hypoxic exposure. However there was no difference between 5 days rest after hypoxic exposure and control group (Table 1). There was no significant difference between each duration of hypoxic exposure and the activity of the brain region.

### 3.2. Western Blot and Immunohistochemistry

**Figure 5:**
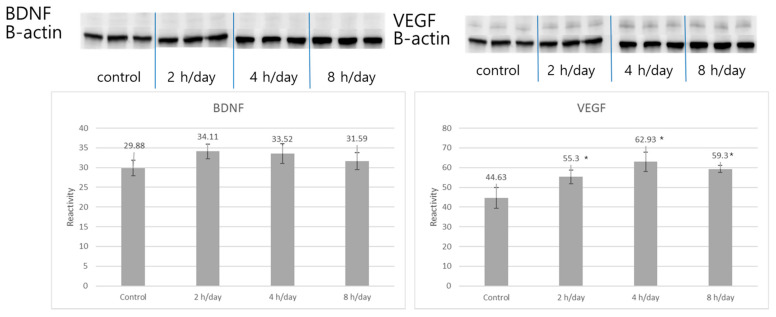
Western blot analysis of VEGF and BDNF expression. The VEGF protein was more reactive in the 2, 4, and 8 h per day groups compared with the control group (*p* = 0.003). However, expression of the BDNF protein showed no difference among the groups (*p* = 0.140). * *p* < 0.05 compared with control group.

**Figure 6:**
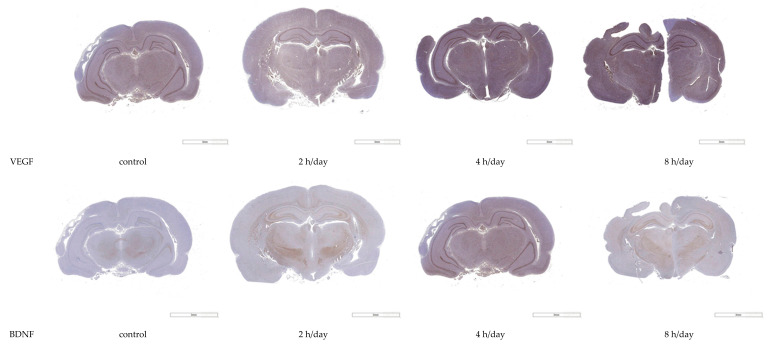

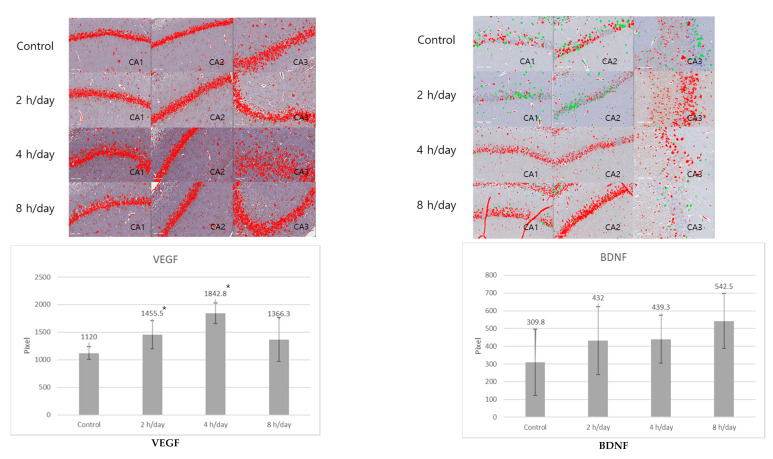
Immunohistochemistry for VEGF and BDNF expression and quantification of immunohistochemistry. Immunohistochemistry analyses of VEGF at hippocampal area revealed significantly greater immunoreactivity in the 2 and 4 h per day groups compared with the control group (*p* = 0.015). * *p* < 0.05 compared with control group.

Western blot analyses and immunohistochemistry with antibodies to VEGF and BDNF were conducted for the hippocampal tissue after the rats had been kept in normal circumstances for five days. The reactivities of the VEGF protein were 44.63±5.28 in the control group, 55.30 ± 3.46 in the 2 h per day group, 62.93 ± 4.91 in the 4 h per day group, and 59.30 ± 1.82 in the 8 h per day group. The reactivities of the BDNF protein were 29.88 ± 2.02 in the control group, 34.11 ± 1.86 in the 2 h per day group, 33.52 ± 2.47 in the 4 h per day group, and 31.59 ± 2.16 in the 8 h per day group. The reactivities of the VEGF protein showed more reactivity in the 2 h per day, 4 h per day, and 8 h per day groups compared with the control group (*p* = 0.003). However, there was no reactivity for BDNF among the groups (*p* = 0.140) (Figure 5). Next, immunochemically stained tissues were quantified using the IHC image analysis toolbox developed based on Image J. The quantification of VEGF immunohistochemistry in the hippocampal area was shown as 1120.0 ± 117.7 in control group, 1455.5 ± 255.8 in the 2 h per day group, 1842.8 ± 135.0 in the 4 h per day group, and 1366.3 ± 399.1 in the 8 h per day group. The pixel of the VEGF positive DAB showed more in the 2 h per day and 4 h per day groups compared with the control group with one-way ANOVA with LSD post hoc test (*p* = 0.015). The quantification of BDNF immunohistochemistry in the hippocampal area was shown as 309.8 ± 186.0 in control group, 432.0 ± 191.9 in the 2 h per day group, 439.3 ± 135.0 in the 4 h per day group, and 542.5 ± 154.1 in the 8 h per day group. There was no significant difference among the groups with one-way ANOVA with LSD post hoc test (*p* = 0.326) (Figure 6). 

### 3.3. Imaging of Brain Activity

**Figure 7:**
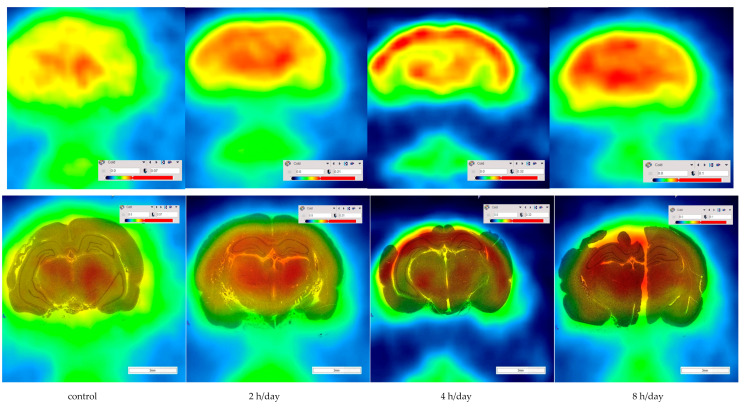
Micro PET-CT imaging on immunohistochemistry. The metabolism in each brain region was shown in each group at 5 days rest after hypoxia.

The micro PET-CT imaging at 5 days rest after hypoxia displayed as color overlays on each immunohistochemistry scan at the level of hippocampus (Figure 7). The pattern of mean SUV in Figure 3 at 5 days rest after hypoxia is presented in Figure 3. 

## 4. Discussion

This research is the first attempt to determine the activities of brain metabolism using animal PET-CT after exposure to intermittent hypoxic conditions according to the duration of hypoxia. In this study, we found elevated brain metabolism after exposure to an intermittent hypoxic condition in rats. Additionally, the brain activity was still elevated after one week’s rest further to us inducing an intermittent hypoxic brain condition in rats. The condition may stimulate cellular vascular proliferation for spontaneous recovery through VEGF elevation. Averaged mean SUV of each hemisphere showed the decrement of brain metabolism at 8 h per day group after five days hypoxic exposure. The quantification of immunohistochemistry also decreased the reactivity of VEGF protein at 8 h per day group compared with 2 and 4 h per day groups. This may suggest that more than 8 h per day 10% hypoxic exposure for 5 days caused irreversible brain damage.

PET scans show the brain metabolism and physiological changes in hypoxic–ischemic brain injury [25]. An ischemic stroke rat model after cardiac arrest showed a reduction in absolute 18F-FDG uptake in all cortical regions. However, significant reductions were not observed in the striatum, hippocampus, and thalamus. There were no changes in the brainstem or cerebellum [26]. Another study reported a correlation between the time course and metabolism after ischemic brain injury following cardiac arrest. The frontal lobe, parietal lobe, brainstem, and cerebellum showed decreased metabolism in PET scans [27]. After inducing a photothrombotic cerebral infarction model, a PET scan showed that 18F-FDG uptake in the infarcted cortex displayed low metabolism at Days 1 and 3, and 18F-FDG uptake in the peri-infarcted area presented high metabolism at Day 7 [28]. Middle cerebral artery occlusion rats displayed low metabolism in the infarcted hemisphere. On the contrary, the contralateral hemisphere showed hypermetabolism [29]. This research showed the metabolism after hypoxic brain injury using PET-CT scans. Additionally, the activity of the brain showed an elevated metabolism after one week under 10% oxygen concentration conditions and maintenance of the metabolism in the 2-h and 4-h hypoxic exposure groups after one week’s rest. Hypoxic conditions could cause hypoxic brain injury and elevate the brain metabolism. The irreversible recovery threshold after hypoxic exposure may be below 8 h of hypoxic exposure under 10% oxygen concentration conditions.

After hypoxic brain injury, various neurotrophins could affect the protection, stabilization, and neuroplasticity of a damaged brain. Among several neurotrophins regulating the neurotrophic pathways related to neurogenesis, the most well-known are BDNF, VEGF, nerve growth factor, neurotrophin-3, insulin-like growth factor-1, and erythropoietin in the adult hippocampus.

VEGF is a neurotrophic factor that may trigger angiogenesis to affect neuroprotection in hypoxic–ischemic brain injury. One of the neuroprotective mechanisms of VEGF has been revealed to be vascular proliferation that supports the migration of neurons after cerebral infarction rat models [30,31]. Many animal studies have reported the effects of exogenously administered VEGF on hypoxic-ischemic brain injury. In a temporary middle cerebral artery occlusion (MCAO) model, the administration of VEGF could reduce brain edema and the volume of cerebral infarction. Interestingly, VEGF causes an immune response and does not cross the blood–brain barrier. The systemic administration of VEGF may stimulate the receptors to increase the permeability of the blood–brain barrier [32]. A hypoxia-induced brain injury may cause an increased density of microvessels, as reported in several previous studies [33,34]. On the other hand, microvessel DNA in the brain did not reveal an alteration after one week of hypoxia, potentially due to an association with microvascular hypertrophy [35]. It is still debatable whether cellular microvascular proliferation may correlate with the recovery of the brain after hypoxic brain injury. In this study, hypoxic conditions induced hypoxic brain injury. The recovery process after brain injury can be proven by the elevation of VEGF expression due to increased angiogenesis. Conclusively, the elevation of brain metabolism may be affected by VEGF expression.

However, there are some limitations to this study. First, it showed a small sample and effect size, and the findings cannot be applied to humans as this was an animal study. No behavioral test was carried out in this research to support the finding of neurological improvement. Another limitation was the absence of histopathologic data induced immediately after the hypoxic period to support the pathologic changes under intermittent hypoxic condition. Further studies are needed to identify therapeutic interventions that can help to recover irreversible hypoxic brain damage.

## 5. Conclusions

This is the first study to attempt to reveal the mechanism related to brain activity after intermittent hypoxic condition-induced brain injury. Vascular endothelial growth factor may be related to the activity of brain metabolism. More than 8 h of hypoxia exposure in rats may cause irreversible brain injury.

## Figures and Tables

**Table 1:** Association of brain region with hypoxic exposure time.

Brain Regions (%ID/g)	Control (*n* = 2)	After Five Days Hypoxic Exposure (^†^ *n* = 6)	*p*-Value	Control (n = 2)	Five Days Rest after Hypoxic Exposure (^†^ *n* = 6)	*p*-Value
Accumbens left	0.00047 ± 0.00004	0.0013 ± 0.00062	* 0.046	0.00054 ± 0.00006	0.00084 ± 0.00042	0.505
Accumbens right	0.00046 ± 0.00001	0.0014 ± 0.00105	* 0.044	0.00055 ± 0.00013	0.00078 ± 0.00035	0.180
Amygdala left	0.00046 ± 0.00003	0.00092 ± 0.0003444	0.096	0.00040 ± 0.00012	0.00067 ± 0.00031	0.505
Amygdala right	0.00042 ± 0.00001	0.00095 ± 0.00055	* 0.046	0.00031 ± 0.00015	0.00063 ± 0.00031	0.505
Striatum left	0.00050 ± 0.00001	0.00133 ± 0.00073	* 0.044	0.00053 ± 0.00004	0.00090 ± 0.00043	0.502
Striatum right	0.00048 ± 0.00004	0.00139 ± 0.00089	* 0.044	0.00048 ± 0.00003	0.00090 ± 0.00044	0.502
Auditory cortex left	0.00047 ± 0.00002	0.00124 ± 0.00071	* 0.044	0.00045 ± 0.00004	0.00080 ± 0.00038	0.502
Auditory cortex right	0.00044 ± 0.00001	0.00117 ± 0.00079	0.180	0.00040 ± 0.00005	0.00073 ± 0.00040	0.502
Cingulate cortex left	0.00049 ± 0.00002	0.00158 ± 0.00122	* 0.046	0.00061 ± 0.00014	0.00091 ± 0.00047	0.505
Cingulate cortex right	0.00048 ± 0.00001	0.00156 ± 0.00123	* 0.046	0.00059 ± 0.00014	0.00090 ± 0.00046	0.505
Entorhinal cortex left	0.00042 ± 0.00002	0.00118 ± 0.00072	* 0.046	0.00045 ± 0.00002	0.00073 ± 0.00033	0.505
Entorhinal cortex right	0.00042 ± 0.00001	0.00126 ± 0.00092	* 0.046	0.00040 ± 0.00004	0.00073 ± 0.00035	0.317
Frontal association cortex left	0.00037 ± 0.00006	0.00109 ± 0.00095	* 0.046	0.00042 ± 0.00013	0.00055 ± 0.00025	0.180
Frontal association cortex right	0.00035 ± 0.00014	0.00129 ± 0.00120	0.096	0.00045 ± 0.00028	0.00057 ± 0.00027	0.317
Insular cortex left	0.00043 ± 0.00002	0.00150 ± 0.00100	* 0.046	0.00052 ± 0.00010	0.00089 ± 0.00040	0.505
Insular cortex right	0.00044 ± 0.00003	0.00173 ± 0.00142	* 0.046	0.00050 ± 0.00005	0.00092 ± 0.00041	0.505
Medial prefrontal cortex left	0.00049 ± 0.00001	0.00137 ± 0.00063	* 0.046	0.00050 ± 0.00001	0.00090 ± 0.00052	0.505
Medial prefrontal cortex right	0.00050 ± 0.00001	0.00137 ± 0.00067	* 0.046	0.00050 ± 0.00001	0.00089 ± 0.00051	0.505
Motor cortex left	0.00043 ± 0.00001	0.00144 ± 0.00122	* 0.044	0.00056 ± 0.00018	0.00077 ± 0.00038	0.505
Motor cortex right	0.00042 ± 0.00003	0.00157 ± 0.00136	* 0.044	0.00054 ± 0.00021	0.00081 ± 0.00042	0.505
Orbitofrontal cortex left	0.00046 ± 0.00003	0.00137 ± 0.00101	* 0.046	0.00051 ± 0.00011	0.00076 ± 0.00034	0.505
Orbitofrontal cortex right	0.00045 ± 0.00001	0.00167 ± 0.00145	* 0.046	0.00061 ± 0.00029	0.00076 ± 0.00035	0.505
Parietal association cortex left	0.00046 ± 0.00001	0.00151 ± 0.00121	* 0.044	0.00055 ± 0.00012	0.00088 ± 0.00042	0.502
Parietal association cortex right	0.00046 ± 0.00001	0.00140 ± 0.00117	0.180	0.00055 ± 0.00012	0.00081 ± 0.00043	0.502
Retrosplenial cortex left	0.00046 ± 0.00001	0.00144 ± 0.00132	* 0.046	0.00053 ± 0.00011	0.00081 ± 0.00031	0.505
Retrosplenial cortex right	0.00047 ± 0.00001	0.00148 ± 0.00140	* 0.046	0.00054 ± 0.00010	0.00079 ± 0.00031	0.505
Somatosensory cortex left	0.00046 ± 0.00001	0.00141 ± 0.00096	* 0.046	0.00053 ± 0.00010	0.00082 ± 0.00044	0.505
Somatosensory cortex right	0.00046 ± 0.00001	0.00132 ± 0.00088	0.182	0.00048 ± 0.00003	0.00082 ± 0.00045	0.505
Visual cortex left	0.00044 ± 0.00001	0.00147 ± 0.00117	* 0.044	0.00054 ± 0.00014	0.00085 ± 0.00039	0.502
Visual cortex right	0.00043 ± 0.00002	0.00146 ± 0.00123	* 0.046	0.00051 ± 0.00014	0.00083 ± 0.00042	0.505
Hippocampus anterodorsal left	0.00043 ± 0.00007	0.00133 ± 0.00107	* 0.046	0.00051 ± 0.00012	0.00081 ± 0.00027	0.317
Hippocampus anterodorsal right	0.00074 ± 0.00008	0.00127 ± 0.00086	* 0.044	0.00048 ± 0.00009	0.00081 ± 0.00031	0.317
Hippocampus posterior left	0.00046 ± 0.00004	0.00110 ± 0.00065	* 0.046	0.00046 ± 0.00004	0.00074 ± 0.00027	0.317
Hippocampus posterior right	0.00047 ± 0.00001	0.00121 ± 0.00089	* 0.046	0.00040 ± 0.00010	0.00074 ± 0.00029	0.505
Hypothalamus left	0.00047 ± 0.00001	0.00100 ± 0.00041	* 0.046	0.00035 ± 0.00016	0.00070 ± 0.00036	0.505
Hypothalamus right	0.00049 ± 0.00001	0.00103 ± 0.00047	* 0.046	0.00036 ± 0.00018	0.00070 ± 0.00035	0.505
Olfactory left	0.00047 ± 0.00007	0.00116 ± 0.00050	* 0.046	0.00046 ± 0.00009	0.00075 ± 0.00039	0.505
Olfactory right	0.00045 ± 0.00003	0.00131 ± 0.00069	* 0.044	0.00046 ± 0.00001	0.00071 ± 0.00036	0.505
Colliculus superior left	0.00050 ± 0.00001	0.00134 ± 0.00106	* 0.040	0.00053 ± 0.00003	0.00079 ± 0.00029	0.495
Colliculus superior right	0.00051 ± 0.00001	0.00128 ± 0.00090	* 0.040	0.00052 ± 0.00001	0.00079 ± 0.00030	0.495
Midbrain left	0.00051 ± 0.00001	0.00132 ± 0.00098	* 0.044	0.00048 ± 0.00005	0.00078 ± 0.00028	0.505
Midbrain right	0.00053 ± 0.00002	0.00133 ± 0.00098	* 0.046	0.00048 ± 0.00010	0.00077 ± 0.00029	0.505
Ventral tegmental area left	0.00053 ± 0.00004	0.00113 ± 0.00050	* 0.046	0.00037 ± 0.00015	0.00077 ± 0.00032	0.505
Ventral tegmental area right	0.00044 ± 0.00001	0.00117 ± 0.00067	* 0.046	0.00040 ± 0.00022	0.00076 ± 0.00033	0.505
Cerebellum GM left	0.00047 ± 0.00001	0.00101 ± 0.00064	* 0.046	0.00039 ± 0.00011	0.00060 ± 0.00025	0.505
Cerebellum GM right	0.00044 ± 0.00001	0.00098 ± 0.00066	* 0.046	0.00038 ± 0.00009	0.00058 ± 0.00025	0.505
Cerebellum WM left	0.00054 ± 0.00001	0.00121 ± 0.00089	* 0.046	0.00041 ± 0.00019	0.00069 ± 0.00030	0.505
Cerebellum WM right	0.00051 ± 0.00001	0.00117 ± 0.00086	* 0.046	0.00041 ± 0.00013	0.00066 ± 0.00030	0.505
Colliculus inferior left	0.00052 ± 0.00007	0.00142 ± 0.00104	* 0.046	0.00051 ± 0.00007	0.00084 ± 0.00037	0.505
Colliculus inferior right	0.00049 ± 0.00003	0.00135 ± 0.00093	* 0.046	0.00054 ± 0.00004	0.00082 ± 0.00035	0.505
Thalamus left	0.00050 ± 0.00003	0.00128 ± 0.00095	* 0.046	0.00052 ± 0.00006	0.00080 ± 0.00027	0.317
Thalamus right	0.00050 ± 0.00003	0.00132 ± 0.00098	* 0.046	0.00050 ± 0.00003	0.00081 ± 0.00029	0.317
Pituitary	0.00042 ± 0.00001	0.00082 ± 0.00027	* 0.046	0.00033 ± 0.00014	0.00056 ± 0.00028	0.505
Cerebellum blood	0.00063 ± 0.00005	0.00146 ± 0.00121	* 0.043	0.00055 ± 0.00015	0.00077 ± 0.00034	0.502
Central canal periaqueductal gray	0.00053 ± 0.00001	0.00134 ± 0.00111	* 0.046	0.00053 ± 0.00001	0.00076 ± 0.00027	0.505
Pons	0.00046 ± 0.00004	0.00092 ± 0.00041	* 0.046	0.00034 ± 0.00022	0.00061 ± 0.00030	0.505
Septum	0.00049 ± 0.00002	0.00125 ± 0.00092	* 0.046	0.00048 ± 0.00001	0.00074 ± 0.00028	0.505
Medulla	0.00057 ± 0.00007	0.00112 ± 0.00055	* 0.046	0.00040 ± 0.00030	0.00070 ± 0.00036	0.505

Values are means ± standard deviations. * *p* < 0.05. **^†^** *n* = 6: *n* = 2 for each 2, 4, 8 h/day treatment. GM: gray matter; WM: white matter.

## Data Availability

The data presented in this study are available on request from the corresponding author.
